# Efficacy and safety of Qishen Yiqi dropping pills combined with modern medicine for coronary heart disease with ischemic heart failure: A systematic review and meta-analysis

**DOI:** 10.1097/MD.0000000000039927

**Published:** 2024-11-01

**Authors:** Qiushan Man, Shijian Chen, Xingyu Li

**Affiliations:** a Department of Cardiology, Chongqing Hospital of Traditional Chinese Medicine, Chongqing, China.

**Keywords:** coronary heart disease, ischemic heart failure, meta-analysis, Qishen Yiqi dropping pills, systematic review

## Abstract

**Background:**

Qishen Yiqi dropping pills combined with modern medicine have been widely used as a treatment for coronary heart disease with ischemic heart failure. Currently, there have been no robust studies addressing the efficacy and safety of Qishen Yiqi dropping pills combined with modern medicine for coronary heart disease with ischemic heart failure. Therefore, this systematic review and meta-analysis are conducted to fill in the gaps mentioned above.

**Methods:**

This systematic review was conducted and reported in accordance with the Preferred Reporting Items for Systematic Reviews and Meta-Analyses guidelines. Relevant studies published from inception to April 17, 2023, in the 5 electronic databases: PubMed, The Cochrane Library, EMBASE, CNKI, and the Wanfang database were comprehensively searched. Weighted mean difference was used as the effect size for the continuous variables. Pooled odd ratios are presented if the results are binary variables. Additionally, we performed subgroup and sensitivity analyses to examine the source of heterogeneity. The funnel plot and the Egger test were used to estimate publication bias.

**Results:**

This meta-analysis included 46 studies involving 5843 participants. There is a significant difference in clinical efficacy, the 6-minute walk test (weighted mean difference = 50.10; 95% CI 28.19 to 72.02; *I^2^* = 98.9%, *P* = .000), the indexes of ultrasonic cardiogram, blood biochemical indexes, and adverse effects (odds ratios = 0.46; 95% CI 0.29 to 0.75; *I^2^* = 35.9%, *P* = .142). The sensitivity analysis and publication bias have demonstrated the robustness of the results (*P* = .702).

**Conclusion:**

Qishen Yiqi dropping pills combined with modern medicine could significantly improve clinical efficacy without incasement adverse effects. Further studies are required to identify the more comprise efficacy and safety results.

## 1. Introduction

Coronary atherosclerotic heart disease is a cardiovascular disease that seriously endangers human health.^[1]^ Due to the long course of the disease and the long-term insufficient blood supply to the myocardium, it is easy to cause myocardial damage, dysfunction, and cardiac complications.^[2]^ Chronic heart failure (CHF) is one of the serious complications of coronary heart disease (CHD), which occurs at the end stage of CHD and is also one of the major causes of death in CHD patients.^[3]^ Heart failure is the terminal stage of heart disease caused by various etiologies.^[4]^ Among them, CHD is a common cause of heart failure which has a high morbidity and mortality.^[5]^ At present, there is no radical cure in clinical practice to delay the progression of the disease.^[6]^ Preclinical heart failure refers to only cardiac structural changes but no obvious clinical manifestations and symptoms.^[3]^ If it is not diagnosed and treated in time, it may gradually develop into refractory end-stage heart failure (heart failure C, D stage), and in severe cases even death.^[4]^

At present, some modern medicines could be used in clinical practice to treat preclinical heart failure.^[2]^ Modern medicines treat CHF in patients with CHD with the aim of lowering blood pressure, diuresis, strengthening the heart, and dilating blood vessels, but the effect is poor and there are certain adverse reactions.^[7]^ Therefore, choosing a more effective treatment plan is the focus of current clinical research. Traditional Chinese medicine believes that the main treatment principle for CHD combined with preclinical heart failure is to activate blood circulation in clinical practice. Chinese medicine treatment not only improves the curative effect but also reduces adverse reactions, highlighting the unique advantages of Chinese medicine.^[8]^

Chinese patent medicine, a type of medicine made from various herbs in a special way in pellets, capsules, or tablets, is widely used in China to treat diseases, especially chronic diseases, because of its convenience and safety. Qishen Yiqi dropping pill is a Chinese patent medicine combined with modern preparation technology based on the traditional theory of traditional Chinese medicine.^[9]^ Recent research has shown that herbal compounds such as salvianolic acid from Danshen (Radix Salviae Miltiorrhizae) can modulate inflammatory signaling pathways such as NF-κB and inhibit the expression of adhesion molecules involved in the progression of atherosclerosis. Additionally, compounds like ginsenosides from Panax Notoginseng (Sanqi) have been found to activate endothelial nitric oxide synthase and promote vasodilation, thereby improving endothelial function and reducing hypertension, a major risk factor for CHD. Qishen Yiqi dropping pills can better improve angina pectoris and cardiac function in patients with CHD without obvious adverse reactions.^[10]^ There are also research results showing that Qishen Yiqi dropping pills can improve the cardiac function and clinical efficacy in CHF patients by inhibiting the expression of inflammatory factors.

Currently, there are no robust studies addressing the efficacy and safety of Qishen Yiqi dropping pills combined with modern medicine for CHD with ischemic heart failure. Therefore, this systematic review and meta-analysis is conducted to further explore the efficacy and the safety of Qishen Yiqi dropping pills combined with modern medicine for CHD with ischemic heart failure and fill in the gaps mentioned above.

## 2. Methods

### 2.1. Literature searching strategy

The literature search was conducted from inception up to Apr 17, 2023, in the 8 electronic databases: PubMed, The Cochrane Library, Web of Science, EMBASE, CNKI, VIP, Wanfang, and CBM. The following terms were used: “Heart Failure, Coronary Disease, Randomized Controlled Trials.” The search was without language restriction. The tailored search strategy for each database and details of the predefined search criteria are provided in Table S1, Supplemental Digital Content, http://links.lww.com/MD/N677. In addition, we also performed a manual search of the references of the relevant studies.

### 2.2. Study selection

After removing duplicates, 2 reviewers independently screened the titles, abstracts, and full texts. Any differences were resolved by consultation between the 2 reviewers (Yi Long and Yingyu Li). Reasons for exclusion were recorded in the full text. Studies, with control groups, investigating the clinical efficacy or safety of Qishen Yiqi dropping pills combined with modern medicine for CHD/ischemic heart failure were included in the meta-analysis. The inclusion criteria were as follows: (1) according to existing diagnostic criteria, patients with a definite diagnosis of CHD with ischemic heart failure, including patients with only coronary artery disease or CHF or concurrent coronary artery disease/CHF, without severity limitation, (2) Qishen Yiqi dropping pills combined with modern medicine was in the intervention group (The main active ingredients of Qishen Yiqi dropping pills are astragaloside IV, danshensu, and salvianolic acids^[11]^), (3) the control group was any treatment without Qishen Yiqi dropping pills, such as beta-blockers/angiotensin receptor neprilysin inhibitors/mineralocorticoid receptor antagonists/sodium-glucose cotransporter-2 inhibitors, and (4) the study type is RCT. Reviews, animal studies, conferences, and other studies without usable data were excluded.

### 2.3. Data extraction

Data extraction was performed independently by 2 reviewers (Qiushan Man and Yi Long) using a standardized form, with disagreement solved by discussion. The following information was extracted: the characteristic of the studies (first author, year of publication, country), the characteristics of participants (sample size, age, gender, etc), efficacy outcomes, including clinical efficacy (significant effect, clinical effect, without effect, overall effective rate according to the guiding principles for clinical research of new drugs in traditional Chinese medicine^[12]^), the 6-minute walk test (6MWT), the indexes of ultrasonic cardiogram (left ventricular ejection fraction [LVEF], etc), blood biochemical indexes (TNF-α, etc), safety outcomes (specific outcomes, any adverse effects). Based on the guiding principles for clinical research of new drugs in traditional Chinese medicine and previous evidence, we classified the outcomes into primary and secondary outcomes. The primary outcomes were the indexes of ultrasonic cardiogram and clinical efficacy. The secondary outcomes were the 6MWT, blood biochemical indexes, and safety outcomes.

### 2.4. Risk of bias assessment

Two independent reviewers (Qiushan Man and Yingyu Li) used a Cochrane tool to assess the risk of bias. Each study was rated as low-, high- or unclear-risk in terms of random sequence generation, allocation concealment, blinding of participants and personnel, blinding of outcome assessment, incomplete outcome data, selective reporting, and other biases. We solved disagreements by discussing with a third investigator (Qiushan Man).

### 2.5. Meta-analysis

This meta-analysis was performed using Stata 14 (Stata Corp., College Station, TX). The weighted mean difference (WMD) was used as the effect size for the continuous variables. The standardized mean difference was used if the data were not consistent in units. Summary odds ratios (OR) were presented if the results were binary variables. The 95% CI and *P*-value were calculated for each effect size. Heterogeneity across studies was evaluated using the *I*^2^. The *I*^2^ value was categorized as low if *I*^2^ was 0% to 25%, moderate if *I*^2^ was 25% to 50%, or high if *I*^2^ was 50% to 90%. Additionally, the Q-statistic was used to assess the presence of heterogeneity. *P*-value by Cochrane’s Q test ≥.05 was considered to indicate no significant heterogeneity among the included studies. If the heterogeneity was low, a fixed-effect model was used. Otherwise, a random-effect model was used. For studies that did not report standard deviation, we filled 0.5 for imputation. Besides, we conducted 2 subgroup analyses. One subgroup analysis for the indexes of ultrasonic cardiogram based on E/A ratio, left ventricular end-diastolic diameter (LVEDD), left ventricular end-diastolic volume (LVEDV), left ventricular end-systolic diameter (LVESD), left ventricular end-systolic volume (LVESV) and LVEF. Another is for blood biochemical indexes based on C-reactive protein (CRP), brain natriuretic peptide (BNP), N-terminal pro-B-type natriuretic peptide (NT-proBNP) and tumor necrosis factor alpha. Additionally, we further performed a sensitivity analysis to assess the robustness of the results. Funnel plots were used to assess potential publication biases. The test of publication bias would not be necessary to analyze if the number of included trials was <10.

We used the grading of recommendations, assessment, development, and evaluations to assess the certainty of evidence of included studies across 5 domains: (1) risk of bias in the individual studies, (2) inconsistency, (3) indirectness, (4) imprecision, and (5) publication bias. We graded the strength of evidence as high, moderate, low, and very low according to grading of recommendations, assessment, development, and evaluations criteria.

## 3. Results

**Figure 1.**
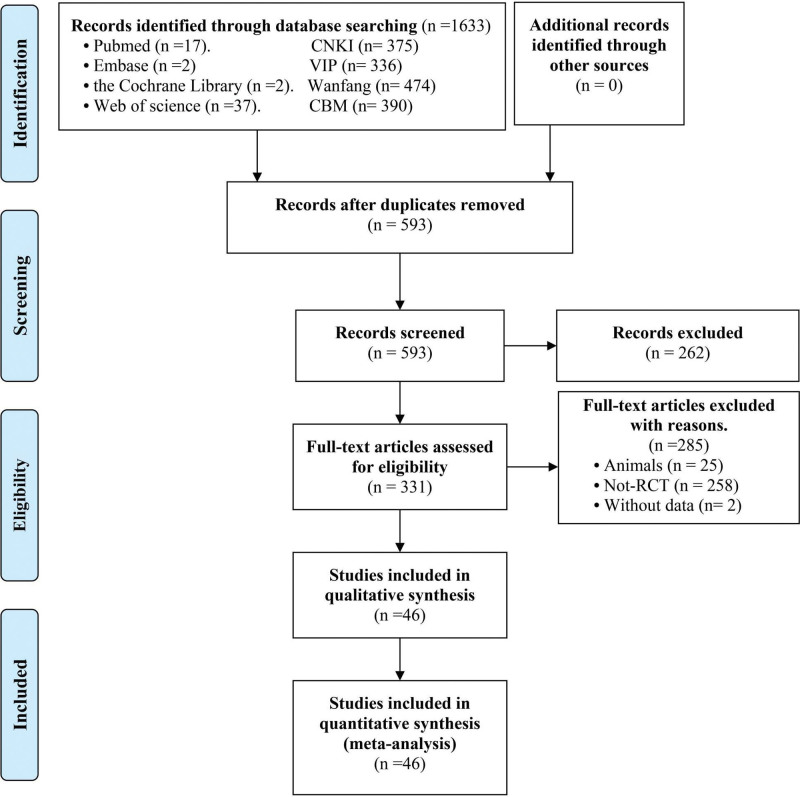
PRISMA flow diagram of screening process. PRISMA = Preferred Reporting Items for Systematic Reviews and Meta-Analyses.

A total of 1633 articles were identified initially. After removing 1040 duplicates, 262 articles were screened out through title and abstract review, leaving 331 articles for further consideration. After excluding 285 studies, the remaining 46 studies were included in the final synthesis (the reasons for exclusion were given in Fig. 1).

A total of 46 studies were included. The included studies were published between 2010 and 2022. A total of 5843 population were included, 2958 of them got the Qishen Yiqi dropping pills combined with modern medicine, and 2885 patients got modern Medicine. Thirty-nine studies used Qishen Yiqi dropping pills at 0.5 g/time, 3 times daily. And 42 studies used regular modern medicine as background therapy/control therapy (Table 1).

**Table 1:** The characteristic of included studies.

Study	Sample size		Gender (male/female)		Age (year)		Disease period (year)		NYHA (1/2/3/4)		Intervention	Background therapy	Administration	GRADE
	I	C	I	C	I	C	I	C	I	C	Duration			
Yunfeng Li (2015)	90	90	112/68		54.6 ± 3.1		4.5 ± 0.5		0/8/19/9		2w	#	$	③high; ④high
Bo Shao (2014)	36	37	22/14	④/13	68.5 ± 9.6	66.7 ± 11.2	NR	NR	0/8/19/9	0/10/16/11	12 w	#	$	①high; ②moderate; ④high
Kaiyuan Zhang (2021)	150	150	96/54	99/51	67.89 ± 9.97	67.02 ± 9.39	3.96 ± 2.93	4.56 ± 3.35	0/30/73/47	0/29/77/44	12 m	#	$	①high; ②moderate; ④high
Yanqing Jia (2010)	29	29	20④/12/17	13/16	63.44 ± 6.20	63.21 ± 6.54	1.09 ± 0.20	1.32 ± 0.23	NR	NR	12 w	Clopidogrel	$	①high; ②moderate; ④high
Jun Zeng (2018)	35	35	19/16	20/15	78.54 ± 6.25	78.56 ± 6.23	1.30 ± 0.35	1.28 ± 0.32	NR	NR	NR	Clopidogrel	$	①high; ②moderate; ④high
Haiqun Hao (2015)	60	60	34/26	34/26	68.4 ± 1.85	65.85 ± 1.7	NR	NR	NR	NR	4w	#	$	④high
Chaozhang Hong (2019)	42	42	23/19	④/18	70.52 ± 4.22	69.54 ± 4.23	NR	NR	0/29/13/0	0/0/28/14	12 w	#	$	①high; ②moderate; ③high; ④high;
Qidong Wang (2018)	48	48	25/23	27/21	64.2 ± 6.1	65.3 ± 6.7	NR	NR	NR	NR	8 w	#	$	①high; ②; ③moderate; ④high
Lingchun Wang (2016)	20	20	20④/9/11	20④/10/10	55.12 ± 4.45	56.58 ± 4.29	NR	NR	NR	NR	3 m	#	&	①high; ②moderate; ③high; ④high
Qifu Che (2018)	50	50	NR	NR	61.43 ± 10.66	64.12 ± 11.34	1.67 ± 0.35	1.72 ± 0.41	0/15/16/19	0/13/17/20	4 w	#	$	①high; ④high
Yayang Liu (2017)	30	30	16/14	17/13	NR	NR	NR	NR	NR	NR	3 m	#	$	①high; ②moderate; ③high; ④high
Qiang Liu (2021)	35	35	22/13	20④/12/23	69.8 ± 8.6	70.7 ± 9.5	5.5 ± 2.8	5.2 ± 2.5	NR	NR	3 m	#	$	①high; ②moderate; ④high
Xiujiang Han (2015)	30	30	20④/12/18	17/13	60.5 ± 10.4	66.2 ± 9.6	3.9士1.3	3.8 ± 1.5	0/8/16/6	0/9/15/6	3 m	#	NR	①high; ④high
Hailian Jia (2012)	40	40	36/44		62.4 ± 8.6		NR	NR	0/30/36/14		8w	#	$	② moderate; ③high; ④high
Tiankun Wu (2015)	120	120	70/50	68/52	70.0 ± 6.1	70.0 ± 7.1	NR	NR	0/12/56/52	0/10/64/46	12 w	#	$	①high; ②moderate; ③high; ④high
Chengyun Yu (2015)	40	40	④/16	23/17	60.5 ± 2.0	60.1 ± 1.1	NR	NR	0/5/20/15	0/6/22/12	8 w	#	$	①high; ②moderate;③high; ④high
Baoli Wu (2018)	50	50	④/26	25/25	65 ± 5.6	64 ± 4.8	NR	NR	0/10/35/5	0/8/36/6	12 w	#	$	①high; ②moderate; ③high; ④high
Ailing Ma (2013)	④	④	NR	NR	NR	NR	NR	NR	NR	NR	NR	#	NR	①moderate;②moderate; ③moderate; ④high
Zhihong Zhao (2018)	42	42	23/19	21/21	62.3 ± 3.8	63.1 ± 2.1	27.3 ± 4.8	④.1 ± 2.4	NR	NR	NR	#	$	①high; ④high
Bingyu Mao (2018)	60	60	35/25	34/26	68.2 ± 9.7	68.1 ± 9.4	8.6 ± 5.1	8.5 ± 5.1	0/16/31/13	0/17/30/13	4 w	#	$	①high; ②moderate; ④high
Mingfeng Gu (2014)	65	65	43/22	38/27	66.8 ± 3.9	67.8 ± 5.6	NR	NR	0/0/53/12	0/0/58/0	2 w	#	$	③high; ④high
Wei Yin (2014)	20	20	20④/11/9	20④/10/10	NR	NR	NR	NR	0/8/7/5	0/9/7/4	6 m	#	$	①high; ②moderate; ③moderate; ④high
Yanxia An (2010)	64	64	36/28	37/27	67 ± 4	68 ± 6	NR	NR	0/0/51/13	0/0/53/11	2 w	#	$	③high; ④high
Dong Wang (2010)	89	76	91/74		57.8		NR	NR	NR	NR	NR	NR	NR	①high; ②;④high
Qingquan Wang (2016)	41	41	48/34		63.5 ± 3.9		7.2 ± 1.4		NR	NR	2w	NR	$	①high; ②; ③high; ④high
Ping Wu (2017)	30	30	16/14	20/20	61.5 ± 2.3	61.8 ± 2.1	NR	NR	NR	NR	3 m	#	$	③high; ④high
Weidong Wang (2011)	40	40	38/42		65.7		7.2		NR	NR	2w	#	$	④high
Lifang Cao (2012)	64	65	36/28	35/30	61 ± 8	61 ± 8	2.6 ± 2.8	2.41.6	0/31/33/0	0/25/40/0	6 w	#	$	④high
Lixia Sun (2018)	60	60	36/④	35/25	61.29 ± 2.35	60.23 ± 2.77	6.29 ± 1.43	6.11 ± 1.54	NR	NR	3 m	#	$	②moderate; ④high
Guowei Lin (2015)	29	27	16/13	20④/12/15	63.22 ± 9.22	63.78 ± 9.95	NR	NR	NR	NR	4 w	#	$	③high; ④high
Yan Xu (2021)	54	54	34/20	33/21	54.7 ± 5.3	55.4 ± 5.4	NR	NR	NR	NR	4 w	#	$	①high; ②moderate; ③high; ④high
Wei Zhang (2019)	47	47	25/22	26/21	63.52 ± 3.27	3.84 ± 0.65	3.95 ± 0.74	3.75 ± 0.82	NR	NR	30 d	#	$	④high
Shuai Wang (2022)	641	584	408/233	359/225	69.02 ± 3.27	66.14 ± 3.27	3.00 ± 3.27	3.00 ± 0.5	0/253/315/73	0/291/237/56	90 d	#	$	④high
Li Yuan (2016)	90	90	47/43	42/48	62/5.8	64/5.2	NR	NR	NR	NR	4 w	#	$	①high; ②moderate; ③high; ④high
Jie Zhang (2012)	79	79	40/39	42/37	60 ± 5.5	62 ± 2.5	NR	NR	0/0/42/37	0/0/38/41	4 w	#	$	①high; ②moderate; ③high; ④high
Yangcheng Shi (2012)	42	42	22/20	23/19	67.5 ± 6.7	66.5 ± 8.2	NR	NR	NR	NR	2 m	#	$	①high; ②moderate; ③high; ④high
Chunhong Qin (2013)	60	54	37/23	32/22	69.5 ± 10.6	68.7 ± 9.8	NR	NR	0/14/30/16	0/14/26/14	8w	#	$	②moderate;③high; ④high
Guihua Zhao (2014)	39	39	④/15	23/16	69.38 ± 10.68	69.71 ± 10.52	NR	NR	0/8/20/11	0/9/18/12	4w	#	$	②;③high; ④high
Qingchi Liao (2017)	40	40	22/18	21/19	55.3 ± 18.8	54.6 ± 16.3	NR	NR	NR	NR	6 m	#	&	①high; ②moderate; ③high; ④high
Yanguang Liu (2019)	27	27	20④/12/15	14/13	64.77 ± 6.49	63.53 ± 7.63	9.04 ± 7.54	8.④±5.21	NR	NR	4 w	#	$	①high; ④high
Yinan Wang (2020)	47	45	27/20	27/18	63.04 ± 11.87	61.78 ± 10.04	7.79 ± 0.63	7.52 ± 0.68	10/7/10/0	10/27/8/0	3 m	#	$	①high; ③high; ④high
Yue Shi (2021)	51	51	27/④	29/22	67.89 ± 5.02	69.02 ± 4.56	9.02 ± 2.34	9.51 ± 2.02	0/12/25/11	0/17/④/10	3 m	#	*	①high; ②moderate; ④high
Hongwen Zhang (2020)	40	40	22/18	23/17	63.09 ± 6.36	63.15 ± 6.46	2.11 ± 0.36	2.08 ± 0.41	0/12/12/16	0/11/12/16	28 d	#	&	①high; ④high
Qiren Li (2022)	60	60	34/26	33/27	55.70 ± 6.28	55.65 ± 6.25	2.47 ± 1.06	2.45 ± 1.05	NR	NR	4 w	#	$	①high; ③high; ④high
Zhenshuang Cui (2020)	35	40	NR	NR	55.16 ± 6.0	52.48 ± 5.3	NR	NR	0/10/25/5	0/6/22/7	6 m	#	$	①high; ②moderate; ③high; ④high
Tengfei Ma (2019)	73	73	36/37	38/35	55.69 ± 4.87	54.51 ± 4.36)	5.71 ± 1.02	5.68 ± 1.05	0/39/34/0	0/40/33/0	3 m	#	*	①high; ④high

# Regular western medicine therapy; $ 0.4 g, tid; & 0.4 g, tid; * 0.52 g, tid.

① The indexes of ultrasonic cardiogram; ② blood biochemical indexes; ③ the 6-minute walk test (6MWT); ④ clinical efficacy; ⑤ safety outcomes.

C = control, D =days, GRADE = grading of recommendations, assessment, development, and evaluations, I = intervention, m = months, NYHA = New York Heart Association Classification, w = weeks.

In this study, quality assessments were conducted on full texts. Table S1, Supplemental Digital Content, http://links.lww.com/MD/N677 presents the results of the Cochrane Collaboration tool for RCT. Overall, 9 studies were rated as high risk of bias. Fourteen studies did not report how to generate the random sequence and were rated as unclear risk of bias. Eight studies were without allocation concealment reporting and rated as unclear risk of bias. Nineteen studies were without blinding details reporting. Another bias of 22 studies was unclear due to funding reporting. No studies had a risk of selective reporting or incomplete outcome data.

### 3.1. The indexes of ultrasonic cardiogram

**Figure 2.**
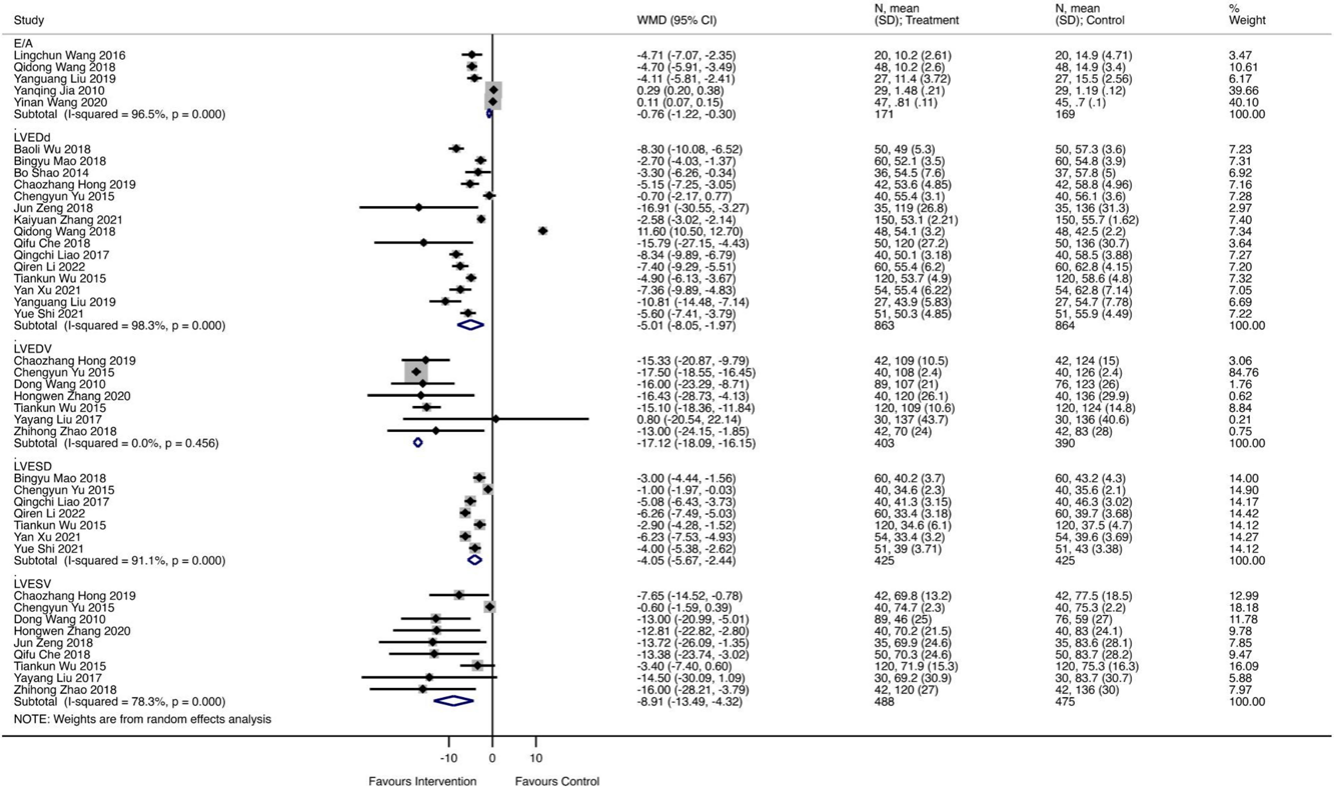
The forest plot of indexes of ultrasonic cardiogram.

**Figure 3.**
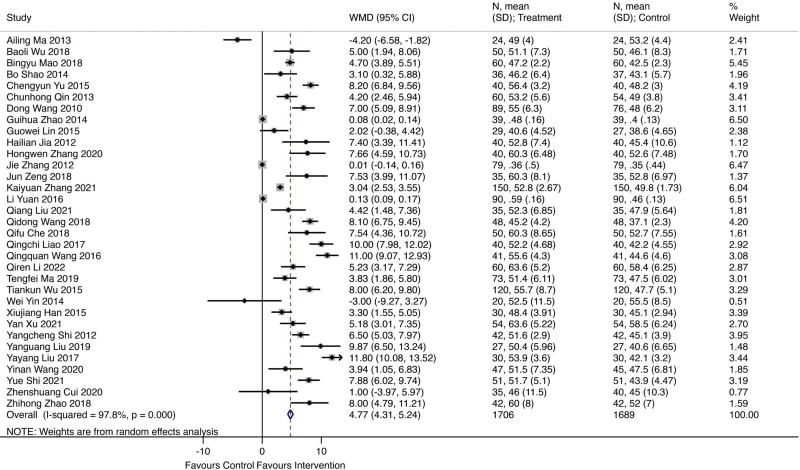
The forest plot of left ventricular ejection fraction (LVEF).

A total of 36 studies involving 2534 patients reported the indexes of ultrasonic cardiograms. Among them, 1274 patients received the combination of Qishen Yiqi dropping pills and modern medicine, and 1260 patients received only regular treatments. Figure 2 showed the forest plot of indexes of ultrasonic cardiogram. Five studies reported an E/A ratio, and results show that Qishen Yiqi dropping pill is associated with significant improvement of E/A ratio (WMD = ‐0.76; 95% CI −1.22 to −0.30; *I^2^* = 96.5%, *P* = .000). Fifteen studies reported LVEDD, and the forest plot showed a significant improvement (WMD = ‐5.01; 95% CI–8.05 to −1.97; *I*^2^ = 98.3%, *P* = .000). A total of 793 patients were involved in LVEDV reporting. There was significant improvement found in the Qishen Yiqi dropping pills combined with the modern medicine group (WMD = ‐17.12; 95% CI ‐18.09 to ‐16.15; *I*^2^ = 0%, *P* = .456). The pooled WMD from 7 studies involving 850 patients showed Qishen Yiqi dropping pills combined with modern medicine intervention improved LVESD for 4.05 value, compared with regular modern medicine therapy (WMD = ‐4.05; 95% CI ‐5.67 to ‐2.44; *I*^2^ = 91.1%, *P* = .000). For LVESV, Qishen Yiqi dropping pills combined with modern medicine intervention could significantly improve LVESV more as 8.91 (WMD = ‐8.91; 95% CI ‐13.49 to ‐4.32; *I*^2^ = 78.3%, *P* = .000). Figure 3 showed the forest plot of LVEF, and the random effect model showed that Qishen Yiqi dropping pills combined with modern medicine intervention could significantly improve LVESV more as 4.77 (WMD = 4.77; 95% CI 4.31 to 5.24; *I*^2^ = 97.8%, *P* = .000).

### 3.2. The 6-minute walk test

**Figure 4.**
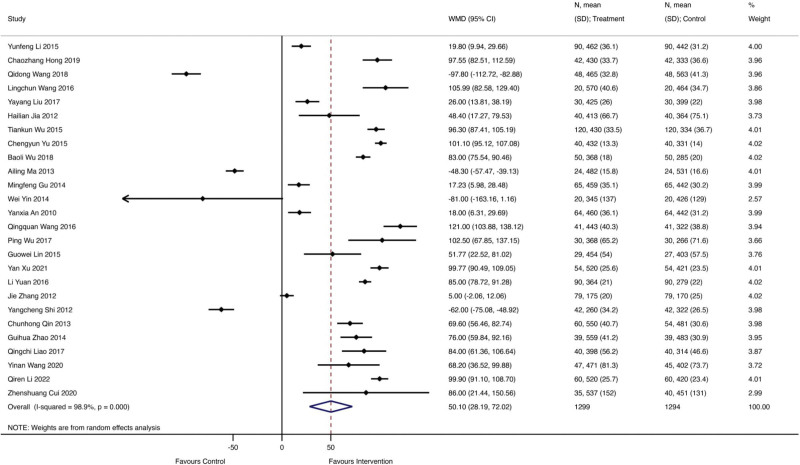
The forest plot of left ventricular ejection fraction (LVEF).

A total of 26 studies involving 1299 patients received Qishen Yiqi dropping pills combined with modern medicine, with 1294 patients receiving regular modern medicine. Figure 4 showed the forest plot of left ventricular ejection fraction (LVEF), and the random effect model showed that Qishen Yiqi dropping pills combined with modern medicine could significantly improve LVESV more as 50.10 (WMD = 50.10; 95% CI 28.19–72.02; *I^2^* = 98.9%, *P* = .000).

### 3.3. Clinical efficacy

**Figure 5.**
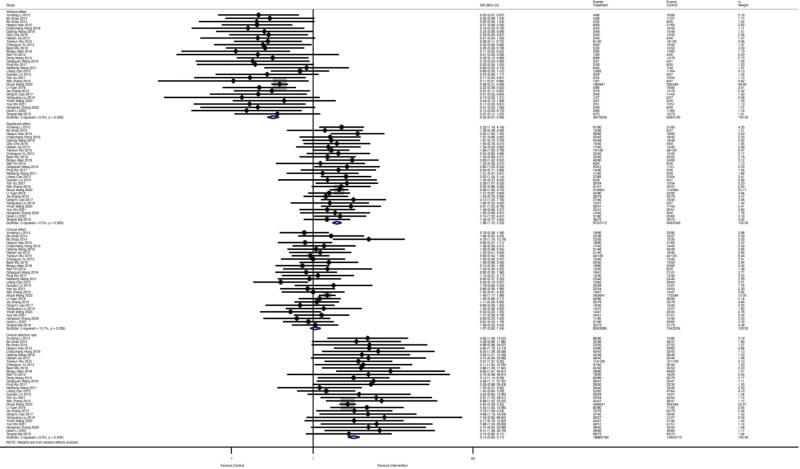
The forest plot of clinical efficacy.

Figure 5 showed the forest plot. A total of 46 studies reported clinical efficacy. A total of 287 patients in the combined treatment group, and 628 patients in the modern medicine group did not report an effect. The random effect model showed that Qishen Yiqi dropping pills combined with modern medicine intervention could significantly reduce the risk without effect as 68% (OR = 0.32; 95% CI 0.27–0.38; *I*^2^ = 98.9%, *P* = .828). There was a significant increase on the risk of significant effect (OR = 1.96; 95% CI 1.72–2.23; *I^2^* = 0%, *P* = .993), clinical effect (OR = 1.07; 95% CI 0.92–1.24; *I*^2^ = 15.7%, *P* = .228) and overall effect (OR = 3.14; 95% CI 2.65 to 3.71; *I*^2^ = 0%, *P* = .934) in Qishen Yiqi dropping pills combined with modern medicine intervention group.

### 3.4. Blood biochemical indexes

**Figure 6.**
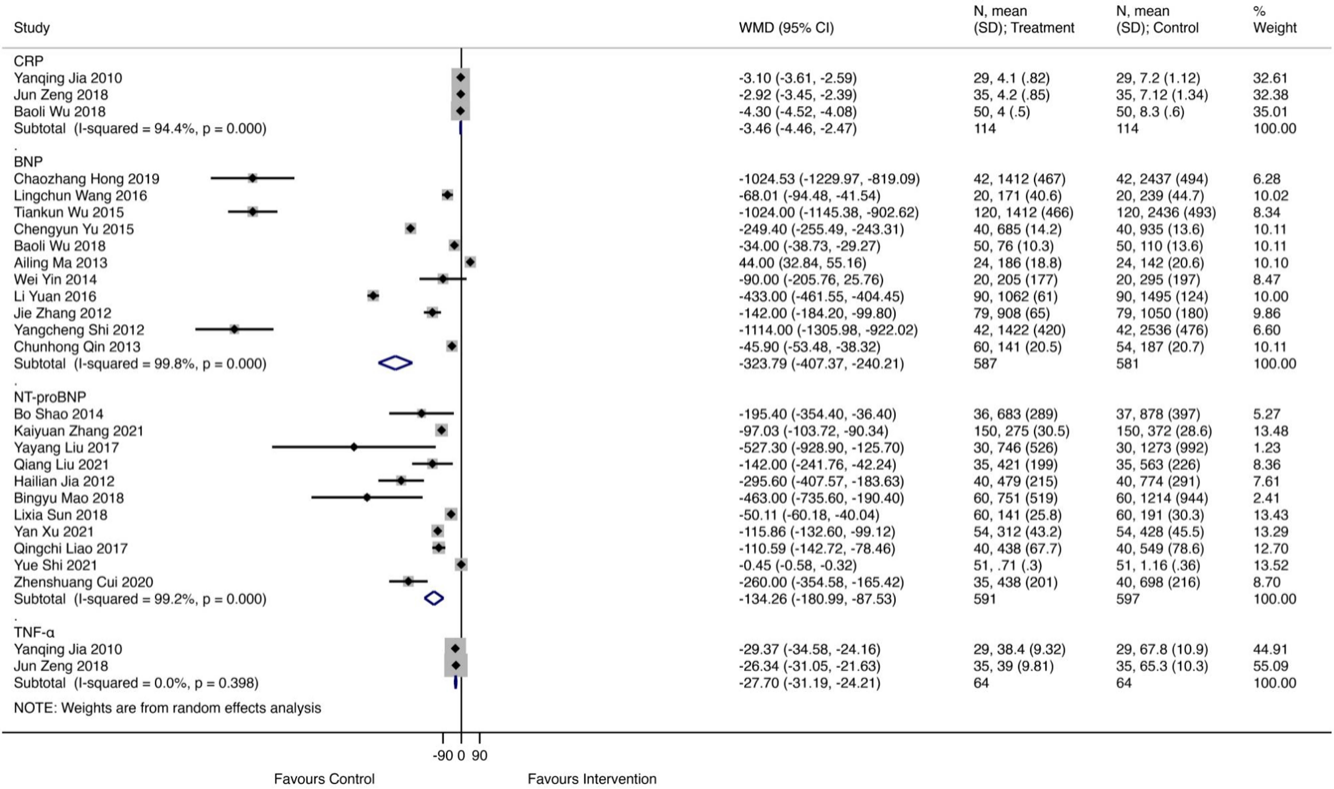
The forest plot of CRP. CRP = C-reactive protein.

There was significant improvement on CRP (WMD = ‐3.46; 95% CI ‐4.46 to ‐2.47; *I*^2^ = 94.4%, *P* = .000, Fig. 6), BNP (WMD = ‐323.79; 95% CI ‐407.37 to ‐240.21; *I*^2^ = 99.8%, *P* = .000), NT-proBNP (WMD = ‐134.26; 95% CI ‐180.99 to ‐87.53; *I*^2^ = 99.2%, *P* = .000) and TNF-a (WMD = ‐27.70; 95% CI ‐31.19 to ‐24.21; *I*^2^ = 0%, *P* = .398) in Qishen Yiqi dropping pills combined with modern medicine group.

### 3.5. Safety outcomes

**Figure 7.**
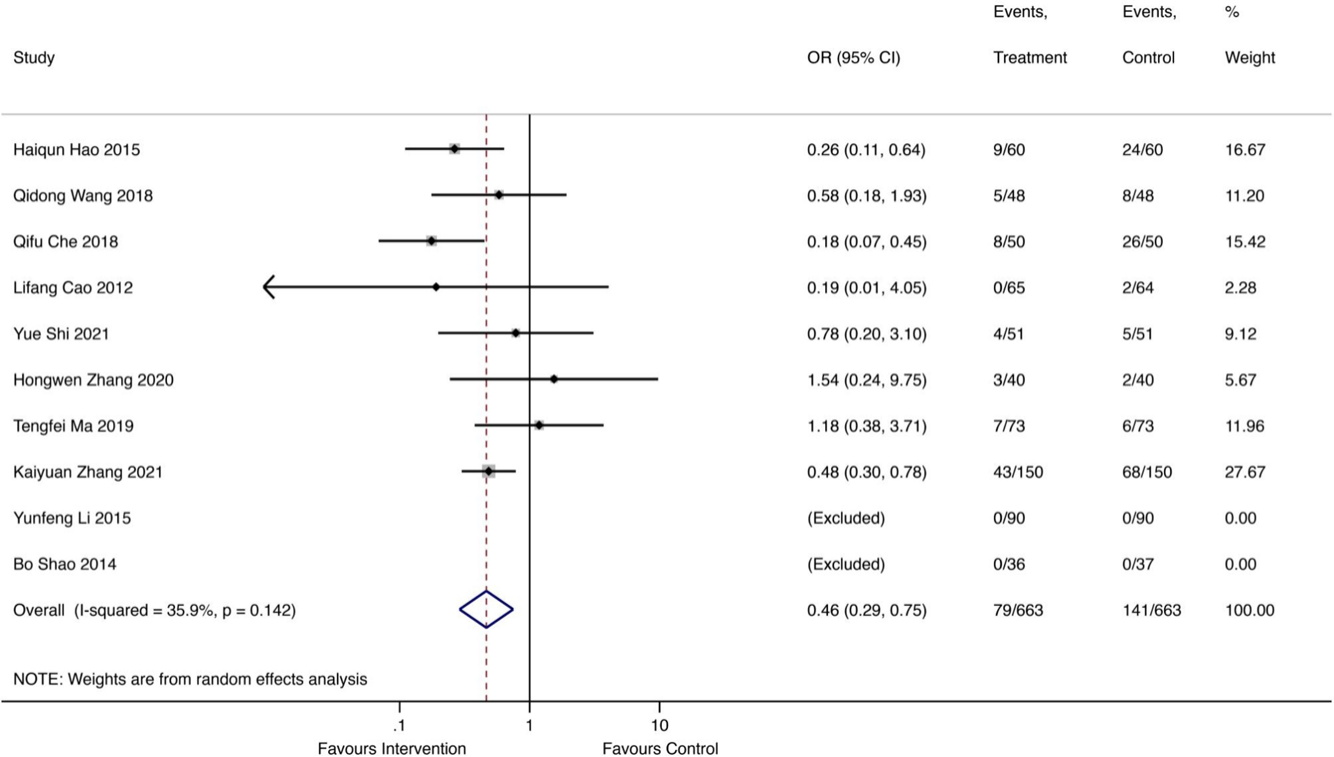
The forest plot of all adverse effects.

A total of 10 studies involving 79 patients received Qishen Yiqi dropping pills combined with modern medicine, with 141 patients receiving regular modern medicine. Figure 7 showed the forest plot of all adverse effects. The random effect model showed that Qishen Yiqi dropping pills combined with modern medicine intervention could significantly decrease the risk of adverse effects (OR = 0.46; 95% CI 0.29 to 0.75; *I*^2^ = 35.9%, *P* = .142).

### 3.6. Subgroup analysis

Our subgroup analysis for the indexes of ultrasonic cardiogram (based on E/A ratio, LVEDD, LVEDV, LVESD, LVESV, and LVEF) and for blood biochemical indexes (based on CRP, BNP, NT-proBNP, and TNF- a) showed that our results are stable.

### 3.7. Publication

Sensitivity analysis was analyzed with the leave-one-out method, the robustness of our results is presented in Figure S1, Supplemental Digital Content, http://links.lww.com/MD/N675. Analysis of the funnel plot of the OR for publication bias suggested the absence of bias because of plot symmetry (Fig. S2, Supplemental Digital Content, http://links.lww.com/MD/N676). The Egger test showed no bias (*P* = .702).

## 4. Discussion

This meta-analysis included 46 studies involving 5843 participants. There is a significant difference in clinical efficacy, the 6MWT, the indexes of ultrasonic cardiogram, blood biochemical indexes, and without incasement on adverse effects. The sensitivity analysis and publication bias have demonstrated the robustness of the results.

Chronic heart failure is a common cardiac syndrome in clinical practice, and it is also the result of the development of various cardiac diseases to the end stage and is the final destination of most cardiovascular diseases.^[13]^ Studies have shown that with the increasing incidence of cardiovascular diseases year by year, the prevalence and mortality of chronic heart failure are also on the rise.^[14]^ Among the underlying heart diseases that lead to chronic heart failure, rheumatic heart disease, which had a large proportion in the past, has shown a downward trend in recent years, while the proportions of hypertension and CHD have increased significantly.^[15]^ Clinically, the traditional treatment of ischemic heart disease and heart failure is to use drugs such as β-blockers to reshape the heart.^[16]^ Modern treatment has reached a plateau, and nondrug treatments (such as cardiac resynchronization therapy) are often expensive and difficult to be widely used in patients.^[17]^ With the continuous development of traditional medicine, the treatment of CHD and heart failure is currently a research hotspot for cardiologists. The treatment principles of traditional Chinese medicine are mainly based on promoting blood circulation, unblocking the veins, and relieving numbness.^[18]^

The Qishen Yiqi dropping pills is a traditional Chinese medicine formulation that has garnered attention for its potential therapeutic effects in cardiovascular diseases, particularly CHD. Key ingredients of Qishen Yiqi dropping pills include Radix Astragali (Huangqi), Radix Salviae Miltiorrhizae (Danshen), and Panax Notoginseng (Sanqi), among others. Modern pharmacological studies have identified various cellular targets of action for these ingredients, including anti-inflammatory, antioxidant, antiplatelet, and vasodilatory effects. The known pharmacology of Qishen Yiqi dropping pills encompasses its multitarget and multipathway actions, which align with the complex nature of CHD pathophysiology. The pharmacologic properties of individual drugs within Qishen Yiqi dropping pills are integral to the results of this manuscript. For instance, Radix Salviae Miltiorrhizae (Danshen) has been extensively studied for its anti-inflammatory and antioxidant properties, which are crucial in mitigating CHD-related inflammation and oxidative stress. Similarly, Panax Notoginseng (Sanqi) has been shown to possess antithrombotic and antiplatelet effects, thereby reducing the risk of thrombotic events in CHD patients. These pharmacological properties collectively contribute to the cardioprotective effects observed with QYDP treatment, as highlighted in the manuscript’s findings.

Qishen Yiqi dropping pills apply modern technology under the guidance of the theory of traditional Chinese medicine, extracting the active ingredients of traditional Chinese medicine Sanqi, Jiangxiang, Astragalus, and Salvia miltiorrhiza for formulation.^[19]^ According to relevant research, Astragalus has good antihypertensive, expansion Vascular, diuretic, and positive inotropic effects, among which the saponin component XGA plays the most important role; the total saponins and notoginseng total glycosides components in Panax notoginseng can significantly increase coronary blood flow and dilate coronary arteries effect, and the 2 can significantly reduce the patient’s arterial pressure and peripheral resistance, thereby significantly improving cardiac function and myocardial blood supply.^[20]^ In 2010, “Qishen Yiqi Dropping Pills Clinical Trial Study on Secondary Prevention of Myocardial Infarction” showed that Qishen Yiqi dropping pills is equivalent to aspirin in the secondary prevention of myocardial infarction. Studies have shown that Astragalus can positively affect muscle strength, dilate blood vessels, and lower blood pressure, and the saponin component XGA plays a major role.^[21]^ Jiang Xiang has a remarkable effect of dispelling blood stasis and promoting blood circulation, which can reduce blood viscosity without significantly affecting the aggregation of red blood cells,^[22]^ while nourishing the heart and calming the nerves. In recent years, it has been found that it has anti-oxidation, anti-inflammation, inhibition of platelet aggregation and coagulation, and reduces blood viscosity, inhibits coagulation, promotes fibrinolysis, prolongs thrombosis and promotes thrombolysis; Panax notoginseng saponins can expand coronary arteries, increase coronary blood flow, and at the same time improve the patient’s myocardial blood supply and heart function. *Astragalus membranaceus* is reused in Qishen Yiqi dropping pills, which not only has the effect of promoting blood circulation, but also can consolidate the surface, while having a good effect on comprehensively improving the heart function of patients.^[23]^

We included the clinical efficacy, the 6MWT, the indexes of ultrasonic cardiogram, and blood biochemical indexes as outcomes. In our results, Qishen Yiqi dropping pills combined with modern medicine is associated with better efficacy and safety. However, this is consistent with the clinical experience. Relevant studies^[18,22,24]^ have found that Qishen Yiqi dropping pills can effectively improve the overall left ventricular function, increase coronary blood flow and oxygen supply in ischemic hearts by regulating energy metabolism pathways, and improve the myocardial structure and function abnormalities caused by ischemia-reperfusion injury. It is also found that Qishen Yiqi dropping pills can effectively inhibit the inflammatory response of patients with myocardial infarction, inhibit the increase of left ventricular end-diastolic diameter, produce anti-ventricular remodeling effects, eliminate free radicals in patients, and promote micro-organisms in the body. It can protect the patient’s extracellular matrix and cardiomyocytes, thereby effectively improving the patient’s heart function.

The strengths of this meta-analysis are as follows: (1) The included studies are with large sample sizes, which increased the confidentiality of the efficacy, and thus these findings are potentially more robust than the previous study. (2) The outcomes are comprehensive enough, including clinical efficacy, and related indexes. (3) Although regular modern medicine was used as control in all included studies, but the difference on components and dose exists in all included studies. This may be the potential source of heterogeneity. (4) Due to all included studies were conducted in China, and the patients were Chinese, the race may influence the application of this study. In the future, more studies from other race will be needed to support the transmission and clinical use of Qishen Yiqi dropping pills.

Our study had several limitations. Firstly, the bias of the included retrospective studies might affect the quality of the evidence. Secondly, the mixed clinical characteristics of included patients may confound the reliability of results, and there is no more precise statement in included studies due to a lack of data. Thirdly, the safety data was insufficient to conduct a quantitative analysis to provide more precise support. Finally, a random effect model is performed based on potential confounding variables, but the heterogeneity will still impact the robustness of the results. Further prospective studies are required to complete an in-depth analysis of the heterogeneity.

Qishen Yiqi dropping pills combined with modern medicine could significantly improve clinical efficacy, the 6MWT, the indexes of ultrasonic cardiogram, blood biochemical indexes, and without incasement on adverse effects. Further studies are required to identify the more comprise efficacy and safety results.

## 5. Conclusion

Qishen Yiqi dropping pills combined with modern medicine could significantly improve clinical efficacy without incasement adverse effects. Further studies are required to identify the more comprise efficacy and safety results.

## Author contributions

**Conceptualization:** Qiushan Man.

**Data curation:** Qiushan Man, Xingyu Li.

**Formal analysis:** Qiushan Man, Shijian Chen, Xingyu Li.

**Project administration:** Shijian Chen.

**Resources:** Qiushan Man, Xingyu Li.

**Writing – original draft:** Qiushan Man, Shijian Chen, Xingyu Li.

**Writing – review & editing:** Qiushan Man, Shijian Chen, Xingyu Li.

## Supplementary Material


