# Supplementary material for: Efficacy and safety of Qishen Yiqi dropping pills combined with modern medicine for coronary heart disease with ischemic heart failure: A systematic review and meta-analysis
Source: Medicine (Baltimore). 2024 Nov 1;103(44):e39927. doi: 10.1097/MD.0000000000039927 (PMC11537573; doi:10.1097/MD.0000000000039927)
Supplement: Supplementary file 1 [file medi-103-e39927-s001.docx]

**Table S1 Search strategy**

| Databases | Search strategy |
| --- | --- |
| PubMed | #11 #10 AND #9 AND #5  #10 (Chinese Patent Drugs ) OR (Qi Shen Yi Qi Di Wan)  #9 #6 OR #7 OR #8  #8 ((Trials, Randomized Clinical) OR (Controlled Clinical Trials, Randomized)) OR (Clinical Trials, Controlled as Topic)  #7 "Controlled Clinical Trials as Topic"[Mesh]  #6 "Randomized Controlled Trials as Topic"[Mesh]  #5 (#1 OR #3) OR (#2 OR #4)  #4 (((((((Cardiac Failure) OR (Heart Decompensation)) OR (Right-Sided Heart Failure)) OR (Right Sided Heart Failure)) OR (Myocardial Failure)) OR (Congestive Heart Failure)) OR (Left-Sided Heart Failure)) OR (Left Sided Heart Failure)  #3 (((Coronary Diseases) OR (Disease, Coronary)) OR (Coronary Heart Disease)) OR (Coronary Heart Diseases)  #2 "Heart Failure"[Mesh]  #1 "Coronary Disease"[Mesh] |
| EMBASE | #7 #5 AND #6  #6 #1 OR #2 OR #3 OR #4  #5 'chinese patent drugs':ti,ab,kw OR 'qi shen yi qi di wan':ti,ab,kw  #4 'coronary disease':ti,ab,kw OR 'multivessel coronary artery disease':ti,ab,kw OR 'coronary artery disease':ti,ab,kw  #3 'coronary disease'/exp  #2 'backward failure, heart':ti,ab,kw OR 'cardiac backward failure':ti,ab,kw OR 'cardiac decompensation':ti,ab,kw OR 'cardiac failure':ti,ab,kw OR 'cardiac incompetence':ti,ab,kw OR 'cardiac insufficiency':ti,ab,kw OR 'cardiac stand still':ti,ab,kw OR 'cardial decompensation':ti,ab,kw OR 'cardial insufficiency':ti,ab,kw OR 'chronic heart failure':ti,ab,kw OR 'chronic heart insufficiency':ti,ab,kw OR 'decompensatio cordis':ti,ab,kw OR 'decompensation, heart':ti,ab,kw OR 'heart backward failure':ti,ab,kw OR 'heart decompensation':ti,ab,kw OR 'heart incompetence':ti,ab,kw OR 'heart insufficiency':ti,ab,kw OR 'insufficientia cardis':ti,ab,kw OR 'myocardial failure':ti,ab,kw OR 'myocardial insufficiency':ti,ab,kw OR 'heart failure':ti,ab,kw  #1 'heart failure'/exp |
| The Cochrane Library | #1 MeSH descriptor: [Heart Failure] explode all trees  #2 MeSH descriptor: [Coronary Disease] explode all trees  #3 MeSH descriptor: [Randomized Controlled Trials as Topic] explode all trees  #4 MeSH descriptor: [Controlled Clinical Trials as Topic] explode all trees  #5 (Cardiac Failure):ti,ab,kw OR (Heart Decompensation):ti,ab,kw OR (Right-Sided Heart Failure):ti,ab,kw OR (Right Sided Heart Failure):ti,ab,kw OR (Myocardial Failure):ti,ab,kw OR (Congestive Heart Failure):ti,ab,kw OR (Left-Sided Heart Failure):ti,ab,kw OR (Left Sided Heart Failure):ti,ab,kw  #6 (Coronary Diseases):ti,ab,kw OR (Disease, Coronary):ti,ab,kw OR (Coronary Heart Disease):ti,ab,kw OR (Coronary Heart Diseases):ti,ab,kw  #7 (Chinese Patent Drugs ):ti,ab,kw OR (Qi Shen Yi Qi Di Wan):ti,ab,kw  #8 (Trials, Randomized Clinical):ti,ab,kw OR (Controlled Clinical Trials, Randomized):ti,ab,kw OR (Clinical Trials, Controlled as Topic):ti,ab,kw  #9 (#1 OR #5) OR (#2 OR #6)  #10 #3 OR #4 OR #8  #11 #9 AND #10 AND #7 |
| Web of science | #1 (((((((TS=(Cardiac Failure) OR TS=(Heart Decompensation)) OR TS=(Right-Sided Heart Failure)) OR TS=(Right Sided Heart Failure)) OR TS=(Myocardial Failure)) OR TS=(Congestive Heart Failure)) OR TS=(Left-Sided Heart Failure)) OR TS=(Left Sided Heart Failure)) OR TS=( Heart Failure)  #2 ((TS=(Coronary Diseases) OR TS=( Coronary Disease)) OR TS=(Coronary Heart Disease)) OR TS=(Coronary Heart Diseases)  #3 TS=(Chinese Patent Drugs ) OR TS=(Qi Shen Yi Qi Di Wan)  #4 ((TS=(Trials, Randomized Clinical) OR TS=(Controlled Clinical Trials, Randomized)) OR TS=(Clinical Trials, Controlled as Topic)) OR TS=(Randomized Controlled Trials as Topic)  #5 #2 OR #1  #6 #5 AND #4 AND #3 |
| CNKI | （主题：心衰+左心衰竭+右心衰竭+心力衰竭（精确）） OR （主题：冠心病+冠状动脉病+冠状粥样硬化性心脏病+冠脉硬化性心脏病+冠脉粥样硬化性疾病+冠状粥样硬化性脏病（精确）） OR （主题：缺血性（精确）） AND （（主题：芪参益气滴丸（精确）） OR （主题：芪参益气丸（精确））） |
| Wanfang | 主题:(心衰 or 左心衰竭 or 右心衰竭 or 心力衰竭 or 冠心病 or 冠状动脉病 or 冠状粥样硬化性心脏病 or 冠脉硬化性心脏病 or 冠脉粥样硬化性疾病 or 冠状粥样硬化性脏病 or 缺血性) AND 主题:(芪参益气滴丸 or 芪参益气丸) |
| CBM | (("冠状粥样硬化性脏病"[常用字段:智能] OR "缺血性"[常用字段:智能]) OR ("心衰"[常用字段:智能] OR "左心衰竭"[常用字段:智能] OR "右心衰竭"[常用字段:智能] OR "心力衰竭"[常用字段:智能] OR "冠心病"[常用字段:智能] OR "冠状动脉病"[常用字段:智能] OR "冠状粥样硬化性心脏病"[常用字段:智能] OR "冠脉硬化性心脏病"[常用字段:智能] OR "冠脉粥样硬化性疾病"[常用字段:智能])) AND ("芪参益气滴丸"[常用字段:智能] OR "芪参益气丸"[常用字段:智能]) |
| VIP | M=(心衰 OR 左心衰竭 OR 右心衰竭 OR 心力衰竭 OR 冠心病 OR 冠状动脉病 OR 冠状粥样硬化性心脏病 OR 冠脉硬化性心脏病 OR 冠脉粥样硬化性疾病 OR 冠状粥样硬化性脏病 OR 缺血性) AND M=(芪参益气滴丸 OR 芪参益气丸) |
