# Supplementary figures and images for: Efficacy and safety of Qishen Yiqi dropping pills combined with modern medicine for coronary heart disease with ischemic heart failure: A systematic review and meta-analysis
Source: Medicine (Baltimore). 2024 Nov 1;103(44):e39927. doi: 10.1097/MD.0000000000039927 (PMC11537573; doi:10.1097/MD.0000000000039927)

# Meta-analysis estimates, given named study is omitted

| Lower CI Limit

○ Estimate

| Upper CI Limit

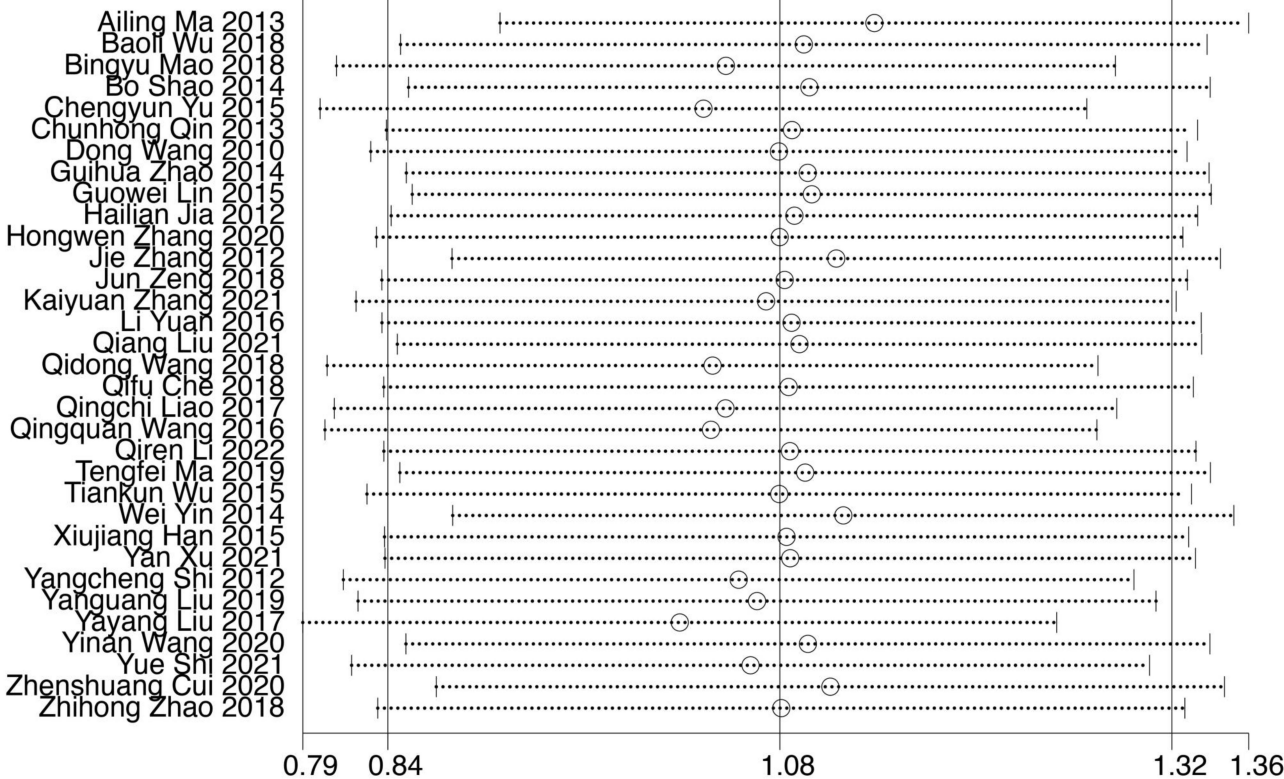

Supplement: Supplementary file 2 [file medi-103-e39927-s002.pdf]

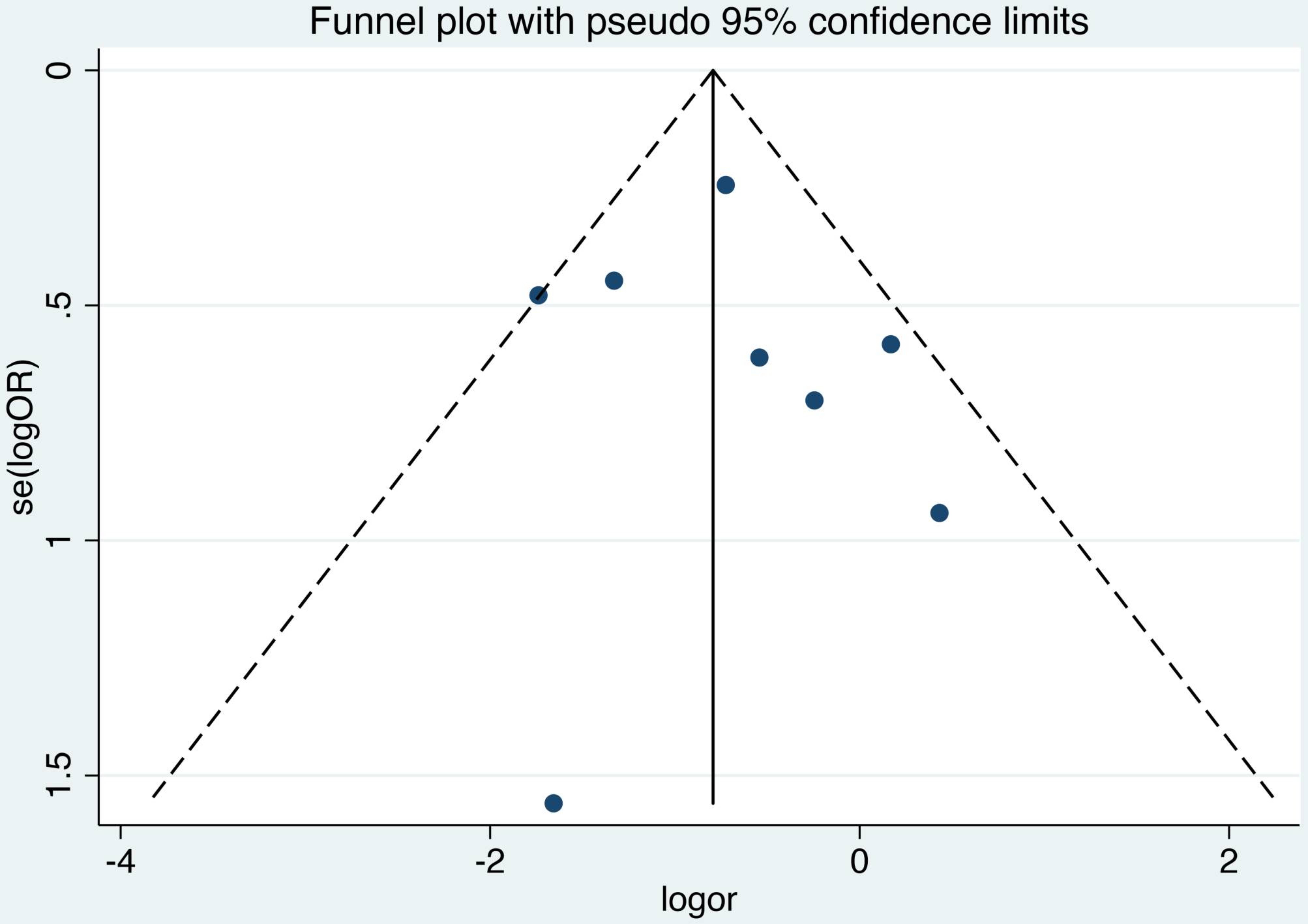

Supplement: Supplementary file 3 [file medi-103-e39927-s003.tif]
